# Fabrication of PEG-PLGA Microparticles with Tunable Sizes for Controlled Drug Release Application

**DOI:** 10.3390/molecules28186679

**Published:** 2023-09-18

**Authors:** Paul Nana Kwame Sagoe, Eduardo José Machado Velázquez, Yohely Maria Espiritusanto, Amelia Gilbert, Thalma Orado, Qiu Wang, Era Jain

**Affiliations:** 1 Department of Biomedical and Chemical Engineering, Bioinspired Syracuse: Institute for Material and Living System, Syracuse University, Syracuse, NY 13244, USA; pnsagoe@syr.edu (P.N.K.S.); yohelymariaa@gmail.com (Y.M.E.); tkorado@syr.edu (T.O.); 2 Department of Biotechnology, SUNY College of Environmental Science and Forestry (ESF), Syracuse, NY 13210, USA; ejmachad@syr.edu; 3 Department of Biomedical Engineering, Rochester Institute of Technology, Rochester, NY 14623, USA; adg7323@rit.edu; 4 School of Education, Syracuse University, Syracuse, NY 13244, USA; wangqiu@syr.edu

**Keywords:** microparticles, PEG, PLGA, coaxial flow, phase separation, drug delivery

## Abstract

Polymeric microparticles of polyethyleneglycol-polylactic acid-*co*-glycolic acid (PEG-PLGA) are widely used as drug carriers for a variety of applications due to their unique characteristics. Although existing techniques for producing polymeric drug carriers offer the possibility of achieving greater production yield across a wide range of sizes, these methods are improbable to precisely tune particle size while upholding uniformity of particle size and morphology, ensuring consistent production yield, maintaining batch-to-batch reproducibility, and improving drug loading capacity. Herein, we developed a novel scalable method for the synthesis of tunable-sized microparticles with improved monodispersity and batch-to-batch reproducibility via the coaxial flow-phase separation technique. The study evaluated the effect of various process parameters on microparticle size and polydispersity, including polymer concentration, stirring rate, surfactant concentration, and the organic/aqueous phase flow rate and volume ratio. The results demonstrated that stirring rate and polymer concentration had the most significant impact on the mean particle size and distribution, whereas surfactant concentration had the most substantial impact on the morphology of particles. In addition to synthesizing microparticles of spherical morphology yielding particle sizes in the range of 5–50 µm across different formulations, we were able to also synthesize several microparticles exhibiting different morphologies and particle concentrations as a demonstration of the tunability and scalability of this method. Notably, by adjusting key determining process parameters, it was possible to achieve microparticle sizes in a comparable range (5–7 µm) for different formulations despite varying the concentration of polymer and volume of polymer solution in the organic phase by an order of magnitude. Finally, by the incorporation of fluorescent dyes as model hydrophilic and hydrophobic drugs, we further demonstrated how polymer amount influences drug loading capacity, encapsulation efficiency, and release kinetics of these microparticles of comparable sizes. Our study provides a framework for fabricating both hydrophobic and hydrophilic drug-loaded microparticles and elucidates the interplay between fabrication parameters and the physicochemical properties of microparticles, thereby offering an itinerary for expanding the applicability of this method for producing polymeric microparticles with desirable characteristics for specific drug delivery applications.

## 1. Introduction

Polymeric microparticles (MPs) are highly regarded as suitable carriers for a wide range of drug delivery applications due to their unique characteristics. Among the different polymeric materials available, polylactide-co-glycolide (PLGA) and poly(ethylene glycol)-poly(lactide-co-glycolide) (PEG-PLGA) copolymers have been widely recognized as ideal options for producing microparticles, owing to their biocompatibility and FDA approval [1,2,3]. While PLGA offers the advantage of controllable drug release due to its favorable degradation properties [3], PEG, due to its hydrophilicity and inert surface charge, can reduce particle aggregation, prolong circulation time, improve cellular uptake, and serve as an anchor for ligand attachment [4]. As a result of these additional benefits, PEG-PLGA is highly desirable for the synthesis of particulate systems for drug delivery applications [5,6].

To date, several methods exist to fabricate PEG-PLGA MPs, the most common being emulsion solvent evaporation, spray drying, electrospray, phase separation, and microfluidics method [7,8]. Depending on the fabrication technique, MPs exhibit distinct size, polydispersity, and morphological characteristics which are crucial to ensuring the stability, encapsulation efficiency, loading capacity, and release of drugs from microparticles [9]. As a result, the biomolecule to be encapsulated and the application for which the MPs are intended remain dependent on the technique chosen for MP synthesis [10,11]. 

While the microfluidic technique allows the fabrication of monodispersed polymeric MPs with precisely controlled size and morphology, its reliance on expensive and complicated instruments makes it challenging for large-scale production [9,12]. On the contrary, despite being quick, easy, and highly scalable, spray drying and solvent extraction and evaporation methods (single and double emulsion) are constrained by their inability to tune and control particle size [11]. As a result, these methods often produce particles with inconsistent batch-to-batch reproducibility, non-uniform size distribution, and low drug loading [5,9,12,13]. Thus, the development of a simple, quick, low-cost, and scalable technique for synthesizing polymeric microparticles with desirable characteristics holds much promise in expanding the use of particulate systems for drug delivery applications.

As opposed to emulsification and solvent extraction-based techniques, phase separation has been demonstrated to be a simple and tunable method for the synthesis of polymeric microparticles with high encapsulation efficiency and narrow size distribution [14]; however, achieving high-scale production is difficult and generating optimal uniform and monodisperse particle size remains a challenge due to the tendency of coarcevate formation caused by particle aggregation [11]. To overcome this limitation, this study presents a novel approach to fabricate PEG-PLGA microparticles of tunable sizes with improved monodispersity and batch-to-batch reproducibility while demonstrating the scalability of particle production yield using a modified phase separation method based on a co-flow technique.

Benefiting from the combination of coflow and phase separation techniques, this modified method offers several advantages including the flexibility of tuning particle size and morphology with uniformity, enhancement of particle production yield, consistency in batch to batch reproducibility, improvement of encapsulation of both hydrophobic and hydrophilic drugs with high loading capacity, and easy and quick processing steps owing to the low dependency on certain conventional microencapsulation steps, such as mechanical agitation, prolonged solvent evaporation, and particle solidification time, which significantly impact particle characteristics [8,15,16]. Moreover, the requirement of low sample volumes, due to the simplicity of our developed coaxial needle construct, makes it beneficial as no special setup and space are needed for sophisticated equipment, thereby leading to a reduction of system footprint [17]. 

We show herein that fabrication parameters had an impact on the microparticle characteristics both independently and in conjunction with each other. Consequently, by varying fabrication parameters such as polymer concentration, surfactant flow rate, and concentration, organic solvent choice, stirring rate, and the volume ratio of the organic phase to the aqueous phase, it was possible to synthesize microparticles of tunable sizes between 5 µm and 50 µm, as well as particles of different morphologies. 

Moreover, the scalability of this method was demonstrated by producing microparticles of comparable sizes yet different particle concentrations through adjustments of either the polymer concentration in the organic phase or the volume ratio of the organic phase to the aqueous phase, a testament to this being evident in the two-fold increase in encapsulation efficiency of rhodamine 6G dye, a model hydrophilic drug. 

## 2. Results and Discussion

### 2.1. Optimization of Experimental Design and Mechanical Parameters 

As a step towards ensuring the reproducibility of this method, we first investigated the impact of major non-fabrication parameters, including mechanical instrumentations and device components, on the characteristics of synthesized microparticles.

Generally, fabrication methods that rely on phase separation to generate emulsion droplets for microparticle synthesis require mechanical agitation, such as stirring, homogenization, or sonication, to create small droplets dispersed in the aqueous phase [18]. Notably, rotor type has been reported to significantly impact the size and distribution of particles as different rotors require specific motors, subsequently influencing the input energy and performance of the stirrer [19,20]. To investigate this, two magnetic stirrers, the RT Basic Series Magnetic Stirrer, Thermo Scientific™, and the Fisherbrand™ Isotemp™ Hot Plate Stirrer, were used for the synthesis of microparticles based on the same formulation conditions. Of interest, while very little difference in morphology was seen for microparticles produced by these two magnetic stirrers, the size of the particles was observed to be impacted, as shown in Figure S1.1 and 1.2 with the RT Basic Series Magnetic Stirrer identified to produce microparticles with smaller and narrow size distribution (9.3 ± 4.0 vs. 6.8 ± 3.1 μm). A potential explanation to this observed difference in size regardless of maintaining all fabrication parameters constant for both stirrers could be due to their respective design, top plate material, wattage requirement, and type of motor. Another process parameter that was investigated was the size of the magnetic stir bar. As indicated in Figure S1.1 and 1.2, an inverse correlation between stir bar size and particle average diameter was observed with the larger stir bar of length 25.4 mm and diameter 8 mm, resulting in a smaller microparticle size and narrow distribution as compared to the smaller stir bar (length and diameter, 12.7 mm and 8 mm) also tested for the same formulation. This resulting difference in size and particle distribution could be attributed to the differences in vortex flow intensity, as a larger magnetic stir bar ensures an appropriate ratio of stir bar length to beaker diameter. Subsequently, influencing the generation of a higher vortex flow intensity facilitates a more vigorous agitation and stronger shear stress necessary to break down large droplets and prevent the coacervation of droplets, as hypothesized in the literature [21]. Thus, the larger stir bar and the RT Basic Series Magnetic Stirrer, Thermo Scientific™, were used for the synthesis of the reference formulation and all subsequent microparticle formulations. This selection was also influenced by the wider range of RT Basic Series stirrer (150–2500 rpm) as compared to the Fisherbrand™ Isotemp™ Hot Plate Stirrer, which is limited to 1500 rpm. Lastly, we also studied the impact of the diameter of the inner needle of the coaxial needle construct, as this will influence the size of the droplet generated. As expected [22], the coaxial needle construct consisting of a bigger inner needle diameter (24 G) resulted in a small yet statistically significant increase in the microparticle size and polydispersity as compared to microparticles synthesized with a smaller inner needle diameter (30 G, which was adopted as a reference parameter for further microparticle synthesis).

Considering these findings, maintaining consistency in both configurational and non-fabrication factors, including the magnetic stirrer type, the dimensions and shape of the stir bar, the beaker or evaporating dish type and size, and the coaxial needle diameter and geometry, remains vital to ensure the reproducibility of this method. Hence, the parameters that yielded microparticles of small size while maintaining consistency in reproducibility across repeated batches were adopted for the establishment of the reference formulation (Figures S1.1 and S1.2).

### 2.2. Preparation and Characterization of Reference Formulation

**Figure 1:**
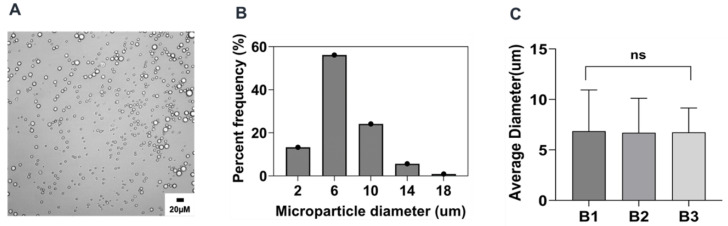
Microparticle size distribution of reference formulation: (**A**) optical micrograph of the microparticle, (**B**) histogram of the microparticle size distribution of a representative batch, (**C**) batch-to-batch comparison of the mean values of three independent batches of the reference formulation. B1: Batch 1, B2: Batch 2, and B3: Batch 3. ns indicates no significance.

In this study, PEG-PLGA microparticles were successfully prepared by a modified phase separation method using a coaxial needle, resulting in co-flow geometry. Since our main objective is to investigate the impact of the individual fabrication parameters on the characteristics of microparticles synthesized via this method, we sought to first prepare microparticles based on a reference formulation against which all other subsequent microparticle formulations were compared. Optical microscopy characterization of these particles revealed a uniform spherical morphology for all microparticles, with a normal particle distribution and an average size of 6.7 ± 3.1 μm, as shown in Figure 1. Notably, no statistically significant difference was observed upon analyzing over 200 particles across three independent batches, thus emphasizing the high reproducibility of this method as illustrated in Figure 1C.

### 2.3. Summary of the Effect of Process Parameters on Microparticle Mean Size and Size Distributions

**Figure 2:**
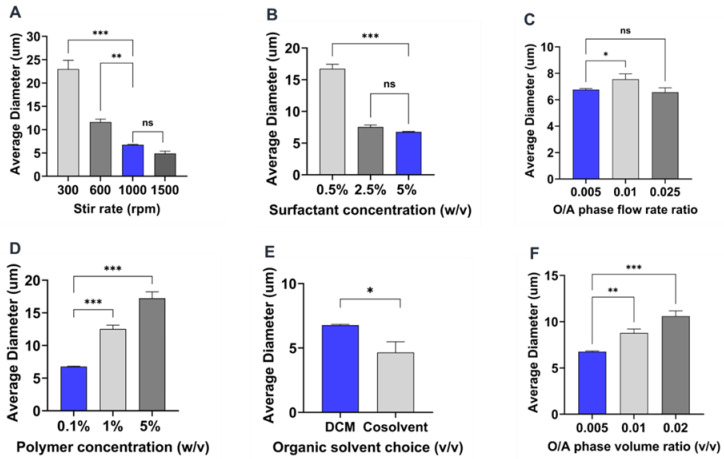
Comparison plots of the influence of individual conditions tested for all process parameters on microparticle size. (**A**–**F**). Blue bars represent each individual reference parameter selected from the reference formulation. One-way ANOVA: * *p* < 0.05, ** *p* < 0.01, and *** *p* < 0.001 Student’s t test: * *p* < 0.05 and ** *p* < 0.01. ns indicates no significance.

A summary of the effect of the process parameters on the PEG-PLGA microparticle formulated by the co-flow phase separation method is provided in Table 1 and Figure 2. Overall, the stirring rate had the highest impact on microparticle size, followed by polymer and surfactant concentration. By varying these parameters, we were able to obtain microparticles ranging in size from 4.7 to 23 μm. The CV values representing polydispersity for each formulation run, calculated by considering all analyzed microparticles across three independent batches as a single batch, remained in the range of 0.33 to 0.53 range. Conversely, the %CV values, determined from averaging the batch-to-batch mean values of three experimental batches of the same formulation, ranged from 1.5% to 17%, thus indicating high reproducibility and narrow particle size distributions. The organic/aqueous phase volume ratio had the most significant impact on reducing polydispersity, but it also led to a slight but significant increase in microparticle sizes under those conditions.

### 2.4. Influence of Process Parameters on Microparticle Characteristics

#### 2.4.1. Effect of Stirring Rate

**Figure 3:**
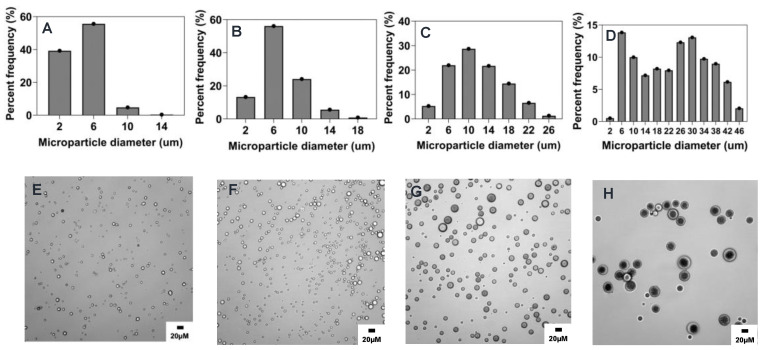
Effect of stirring rate (rpm) on microparticle size and distributions. Optical micrograph and histogram of particle size distribution: (**A**,**E**) 1500 RPM, (**B**,**F**) 1000 RPM, (**C**,**G**) 600 RPM, and (**D**,**H**) 300 RPM.

Generally, as the stirring rate increases, the shear energy generated by the magnetic stirrer also increases, thereby causing large droplets of the polymer phase to be broken into smaller droplets [23]. Consistent with previous studies [24,25,26], an inverse relationship was observed between stirring rate and mean particle size. As the stirring rate was increased from 300 rpm to 1500 rpm, the particle size of the microparticles decreased from 23 ± 1.9 μm to 4.9 ± 0.5 μm as shown in Table 1 and Figure 2A and Figure 3. On comparing to the reference formulation, we did not find a statistically significant difference between microparticle mean sizes made using 1000 rpm or 1500 rpm (Figure 2A). However, there was a statistically significant difference when comparing the reference formulation to the 300 rpm (*p* < 0.01) or 600 rpm (*p* < 0.05), which had higher mean microparticle sizes (Figure 2A and Figure 3C, D and Table 1). The observation in the particle size distribution also aligns with similar reports showing that increasing the stirring rate improves the mixture and diffusion of the phases, resulting in microparticles with a relatively narrow particle size distribution [24]. For instance, while 90% of all microparticles were in the size range of 2–6 μm when made at 1500 rpm, only ~30 and ~15% of microparticles were in that size range when fabricated at 600 and 300 rpm, respectively (Figure 3A,C,D).

#### 2.4.2. Effect of Surfactant Concentration

**Figure 4:**
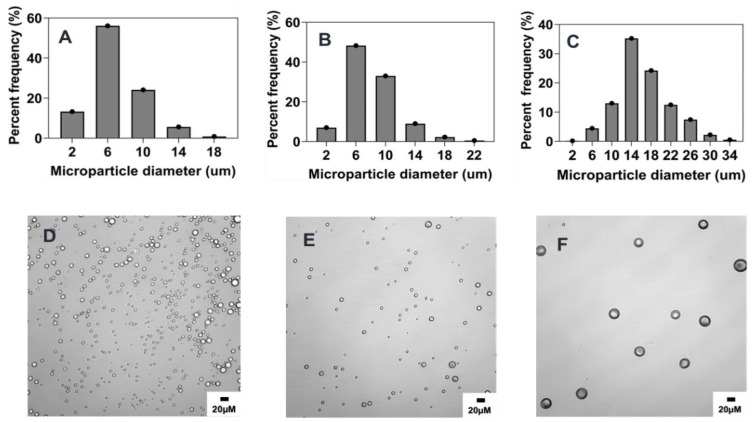
Effect of surfactant (PVA) concentration (% *w*/*v*) on microparticle size and distributions. Optical micrograph and histogram of particle size distribution: (**A**,**D**) 5% *w*/*v*, (**B**,**E**) 2.5% *w*/*v*, (**C**,**F**) 0.5% *w*/*v*.

Several previous studies have shown that surfactant concentration in the aqueous phase has a significant effect on the size and size distribution of the microparticles [6,23,26]. Generally, an increase in the concentration of surfactant has been shown to cause a decrease in microparticle size [16,27,28]. In this study, the concentration of PVA in the aqueous phase was identified to have a negative correlation with the average size of microparticles. Hence, as PVA concentration was increased from 0.5% *w*/*v* to 5% *w*/*v*, a corresponding decrease in size from 16.7 ± 0.7 μm to 6.8 ± 0.1 μm (*p* < 0.001) was observed, as indicated in Figure 2B and Figure 4 and Table 1. Similar effects of increasing surfactant concentration were also seen when the polymer concentration was changed to 1% *w*/*v* and PVA concentration was varied from 0.5 to 10% *w*/*v* (Supplementary Information; Figure S2). At higher concentrations of PVA, the interfacial tension between the organic and aqueous phases decreases [29,30], leading to the stabilization of the emulsion against droplet coalescence, hence the observed finding. Interestingly, there was no significant size difference between microparticles synthesized with 2.5% *w*/*v* and 5% *w*/*v* PVA concentrations despite the former being slightly larger (Figure 2B and Figure 4). This could be due to the similarity in the emulsification effect induced by these two concentrations of PVA, as it has been reported that the concentration of surfactants with comparable viscosity produces a similar effect against particle coalescence [25,30]. Although the particle mean particle size was not significantly different between the two formulations, the particle distribution showed that about ~76% and ~60% of particles were in the size range of 2–6 μm when made using 5% or 2.5% *w*/*v* PVA, respectively (Figure 4). Another observed effect worth mentioning is the impact of the concentration of PVA on the morphology of the microparticles [31]. As shown in Figure 4D,F**,** microparticles synthesized with 5% *w*/*v* PVA concentration were characterized by a high degree of smoothness and sphericity, while the surface of microparticles produced by 0.5% *w*/*v* was observed to be rough and wrinkled. Other formulations with increased PVA concentration at 10% *w*/*v* and 0.5% *w*/*v* also show the emergence of an oval shape (aspect ratio ~1.7) irregular morphology, respectively (Figure S2).

#### 2.4.3. Effect of Organic and Aqueous Phase Flow Rate Ratio

**Figure 5:**
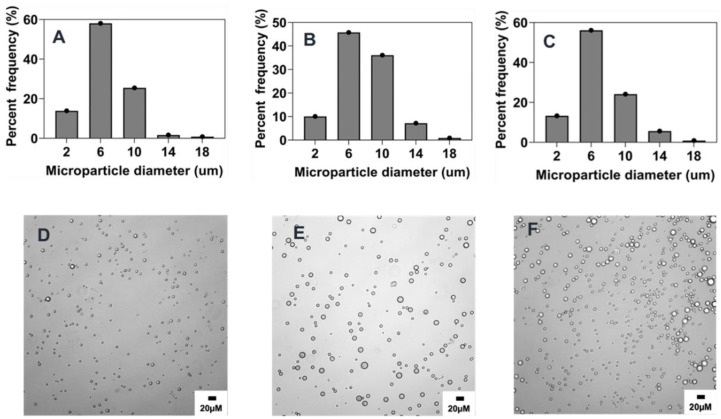
Effect of organic/aqueous phase flow rate ratio on microparticle size and distributions. Optical micrograph and histogram of particle size distribution: (**A**,**D**) 0.025, (**B**,**E**) 0.01, (**C**,**F**) 0.005.

As established in the literature, fabrication methods based on co-flow technology to generate droplets are influenced by the flow rates of the organic and aqueous phases [22,32,33,34,35]. In this study, the influence of the flow rate ratio of the organic to aqueous phase was investigated across three different ratios (0.005, 0.01, and 0.025), as presented in Figure 2C and Figure 5 and Table 1. In agreement with previous findings [12,22,34], a slight increase in microparticle size from 6.8 ± 0.1 µm to 7.6 ± 0.4 µm was observed when the flow rate ratio was increased from 0.005 to 0.01, as a generally higher flow rate ratio due to a lower flow rate of the aqueous phase results in a faster production of polymer droplets, thereby increasing the tendency of particle coalescence. On the contrary, a further increase in the flow rate ratio to 0.025 resulted in a slight decrease in size to 6.6 ± 0.3 µm (Figure 2C and Figure 5). The lack of significance observed in these formulations may be attributed to the overriding influence of dominant parameters that determine particle size, such as stir rate and polymer concentration. Another possible explanation for this observation could be the stabilizing effect of the PVA in the collecting vessel prior to initiating the co-flow. This may have minimized the impact of a higher flow rate ratio despite the dispersed phase having higher flow rates that would typically result in faster generation of polymer droplets in the emulsion.

#### 2.4.4. Effect of Polymer Concentration

**Figure 6:**
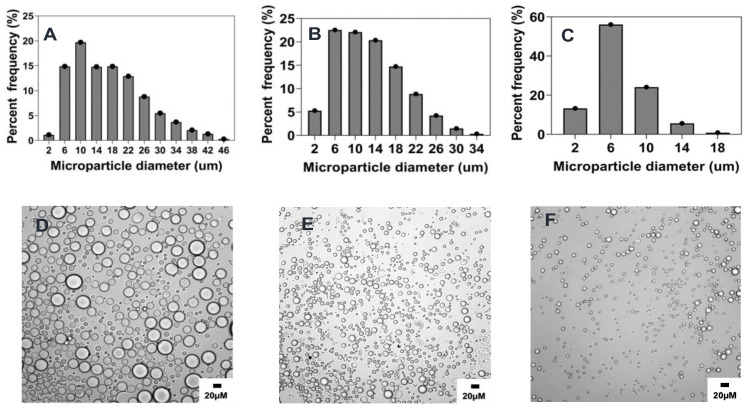
Effect of polymer concentration (%*w*/*v*) on microparticle size and distributions. Optical micrograph and histogram of particle size distribution: (**A**,**D**) 5% *w*/*v*, (**B**,**E**) 1% *w*/*v*, (**C**,**F**) 0.1% *w*/*v*.

Increasing polymer concentration has been shown to increase the size of particles [23,34,36]. This observation can be explained by the heightened viscosity of the organic phase caused by the increased concentration of the polymer solution, making it challenging to generate small emulsion droplets and ultimately leading to an increase in particle size [6,37]. Consistent with previous literature, this study’s findings demonstrate that the concentration of PEG-PLGA in the organic phase has a significant impact on microparticle size. As shown in Figure 2D and Figure 6 and Table 1, increasing the concentration of PEG-PLGA in the organic phase from 0.1% *w*/*v* (reference formulation) to 5% *w*/*v* was accompanied by a corresponding increase in the mean particle size from 6.8 ± 0.1 µm to 17.2 ± 1 µm) (*p* < 0.001, Figure 2D). We also observed wider size distribution with increasing polymer concentration, where formulation made using 0.1% polymer concentration had ~76% of particles below 6 μm while formulations of 5% polymer concentration had only ~17% of particles in that size range (Figure 6A,C).

#### 2.4.5. Effect of Organic Solvent Choice

**Figure 7:**
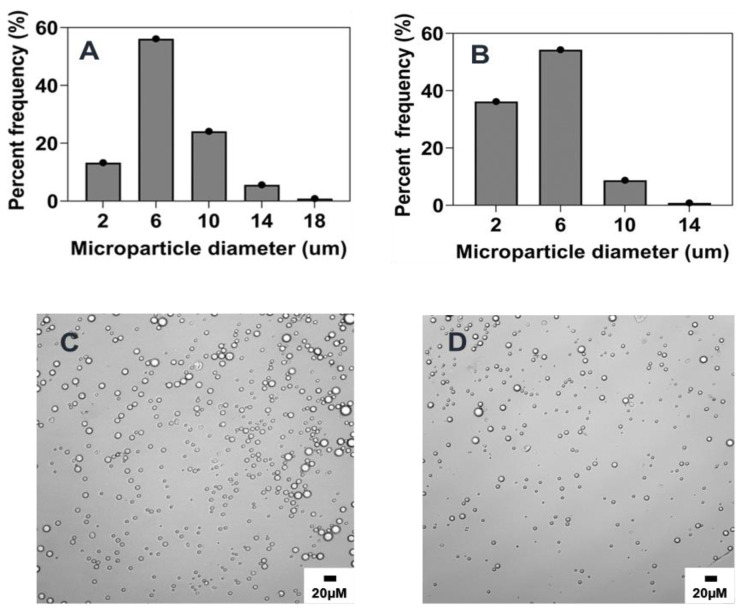
Effect of organic solvent choice on microparticle size and distributions. Optical micrograph and histogram of particle size distribution: (**A**,**C**) DCM, (**B**,**D**) ACN: DCM (25:75 *v*/*v*).

Generally, organic solvents with increased solubility in water generate particles with smaller particle sizes and narrower size distribution [38]. Thus, it was anticipated that the introduction of acetonitrile (ACN), a more polar solvent in DCM, would increase the solubility of the resulting organic phase in the aqueous phase, thereby leading to the generation of microparticles with smaller size. As shown compared to the reference formulation (Figure 2E and Figure 7 and Table 1), the size of microparticles decreased from 6.8 ± 0.1 µm to 4.7 ± 0.8 µm (*p* < 0.05) upon adding 25% *v*/*v* ACN as a cosolvent to pure DCM. There is also a minor difference in particle size distributions with formulations made in ACN-DCM, which have about ~38% of microparticles below the size of 2 μm, while in DCM, only ~16% of microparticles are below 2 μm. This finding aligns with previous studies that report an increase in microparticle size synthesized using DCM as the organic phase solvent as compared to ethyl acetate, a moderately polar solvent similar to ACN [16,27].

#### 2.4.6. Effect of Organic to Aqueous Phase Volume Ratio

**Figure 8:**
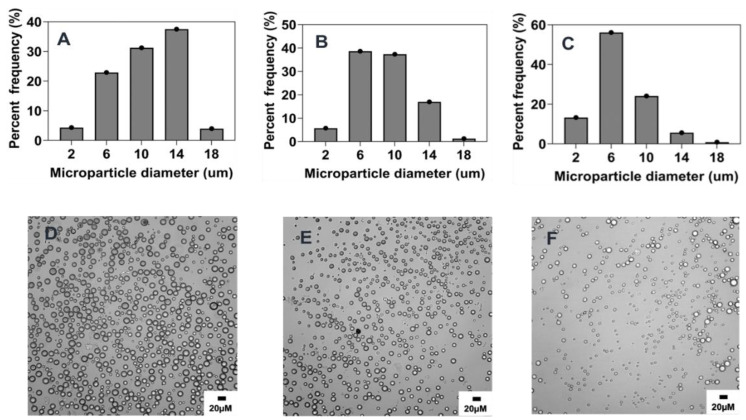
Effect of organic/aqueous phase volume ratio on microparticle size and distributions. Optical micrograph and histogram of particle size distribution: (**A**,**D**) 0.02% *v*/*v*, (**B**,**E**) 0.01% *v*/*v*, (**C**,**F**) 0.005% *v*/*v*.

The size of the microparticles has also been described to be influenced by the variation of the volume ratio of both the organic and aqueous phases [27,30,39]. For this investigation, the volume of the aqueous phase was maintained at a constant 20 mL, and the volume of the dispersed phase was increased from 0.1 mL to 0.4 mL, constituting an organic: aqueous phase volume ratio of 0.005% *v*/*v* and 0.02% respectively. As shown in Figure 8 and Figure 2F and Table 1, the size of the microparticles increased (*p* < 0.01) from 6.8 ± 0.1 µm to 10.6 ± 0.6 µm, with a corresponding elevation in particle concentration or number of particles formed as the phase volume ratio was increased. This observation aligns with similar findings reporting the role of the viscosity of the emulsion due to an increase in the dispersed phase as a justification for the increase in particle size [20,30]. Other explanations could be the increase in the tendency of collision and coalescence among the dispersed oil droplets as a higher phase volume ratio allows for a lesser distance between the aqueous and organic phases, leading to poor phase separation [40,41].

### 2.5. Scalability and Tunability of Co-Flow Phase Separation Method

While phase separation has been reported as a tunable method, achieving high-scale production remains challenging [11,42]. Here, by utilizing a combination of specific determining fabrication parameters that have been discussed in this study to impact microparticle size and concentration, we demonstrate that microparticles of comparable size to the reference formulation can be synthesized while achieving high scale-up production without compromising particle characteristics. Here, we varied the three parameters surfactant (PVA) concentration, stir rate, and polymer concentration, which significantly affected particle size simultaneously to obtain particles of similar size and narrower polydispersity. Our results demonstrate that careful combination and variation of these parameters can allow us to obtain particles with higher yields, desired particle size, and narrow particle size distribution.

#### 2.5.1. Combined Effect of Increasing Phase Volume Ratio, Surfactant Concentration and Stir Rate

**Figure 9:**
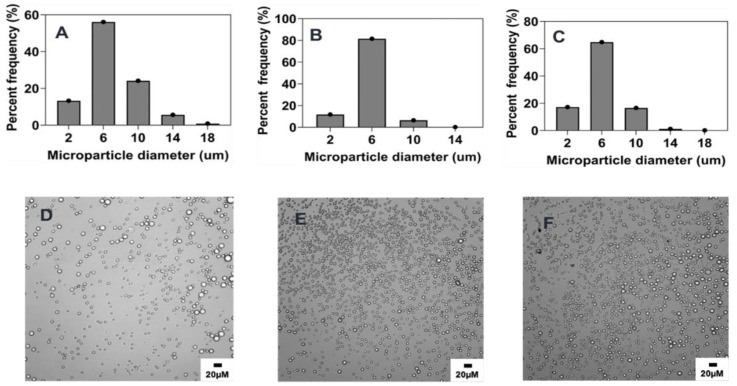
Optical micrograph and histogram of the particle size distribution of scaled-up microparticles produced by different PVR: (**A**,**D**) 0.005% *v*/*v*, (**B**,**E**) 0.01% *v*/*v*, (**C**,**F**) 0.02% *v*/*v*.

While an increase in the organic/aqueous phase volume ratio has been shown to increase the number of particles generated, this effect also leads to a size increase of particles, as supported by our data (Figure 7 and Figure 8F) and others [20,43]. As opposed to this, the concentration of surfactant (PVA) and the stirring rate of the emulsion have both been identified to have an inverse correlation to particle size (20, 27–31). Thus, to keep microparticle size in a comparable range to the reference formulation, increasing PVA concentration and stir rate was adopted as a strategy to control the potential increase in particle size owing to the subsequent increase of the organic/aqueous phase volume ratio. As depicted in Table 2 and Figure 9, when the volume ratio of the organic to aqueous phase was increased, along with higher PVA concentration and stir rate of the emulsion, particles with similar morphology and size to the reference formulation were synthesized. Notably, these particles exhibited reduced polydispersity, narrower size distribution, and increased particle concentration (Figure 9).

#### 2.5.2. Combined Effect of Increasing Polymer Concentration, Surfactant Concentration, and Stir Rate

**Figure 10:**
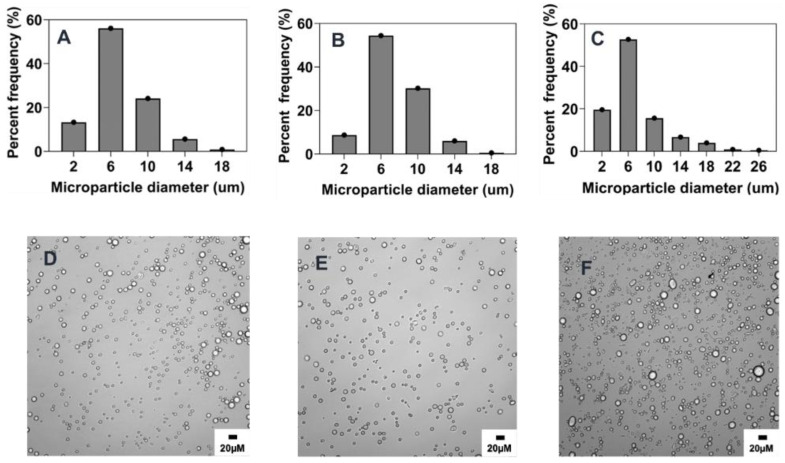
Optical micrograph and histogram of the particle size distribution of scaled-up microparticles produced by increased polymer concentration: 0.1% *w*/*v* (**A**,**D**), 1% *w*/*v* (**B**,**E**), 5% *w*/*v* (**C**,**F**).

Generally, since microparticles are generated by oil droplets owing to the polymer dissolution in the organic phase, one will expect a higher polymer concentration to correspond to an increase in oil droplets, subsequently promoting the production of more microparticles. While this is true, the formation of more oil droplets due to increased polymer concentration also increases the tendency of particle coalescence that ultimately leads to an increase in particle size, as shown by our data (Figure 5 and Figure 8D) and found in the literature [5,6]. To avoid this effect and foster the synthesis of microparticles with properties comparable to the reference formulation, an increase in PVA concentration and the emulsion stir rate speed was adopted as similarly investigated for the scalability achieved via increasing the organic/aqueous phase volume ratio. As shown in Figure 10, increasing polymer concentration with high PVA concentration and stir rate resulted in the successful tuning of the microparticles to an approximate size of ~7 μm while achieving increased microparticle concentration. As reported in the literature, usually increasing polymer concentration increases microparticle size, which reduces the range of applications [11,30,42]. We hereby present a method whereby controlling PVA concentration and stir rate led to the formation of particles of similar size irrespective of the polymer concentration. More specifically, we attained microparticles measuring around ~7 μm in size across three distinct formulations, with the formulation based on the highest polymer concentration resulting in an increase in particle polydispersity as shown in Table 3.

To further illustrate the tunability and the microparticle size ranges that can be obtained using this method with high polymer concentration, we also tested the combined effect of increasing polymer and PVA concentration with a lower stir rate to examine the effect of the surfactant concentration. Our result indicates that microparticle size up to ~40 μm can be obtained at a lower stir rate and higher concentration of PVA, which also influences microparticle morphology (Figure S2). Lastly, to demonstrate the versatility of this method to obtain a range of microparticles, we simultaneously varied several process parameters and obtained microparticles in different size ranges, including ~50, 76, 100, and 114 μm (Supplementary data Table S3 and Figure S4).

### 2.6. Encapsulation of Model Dyes in PEG-PLGA Microparticles

To showcase the potential application of microparticles generated through this method for controlled drug delivery, hydrophilic rhodamine 6G and hydrophobic coumarin 6, were incorporated as model drugs into the organic phase at a theoretical loading of 1% *v*/*v* dye concentration. Thus, by maintaining all process parameters at the levels specified by the reference formulation, Rho6G and Coum6-loaded microparticles were prepared. As expected, the encapsulation efficiency and drug loading of coumarin 6, a hydrophobic dye, were found to be approximately two times higher than that of the hydrophilic rhodamine in the microparticles, as shown in Table 4. We also observed a small but significant increase (*p* < 0.05) in microparticle size with encapsulation of coumarin, while no such significant difference in size was found in rhodamine-loaded microparticles (Figure S3).

**Figure 11:**
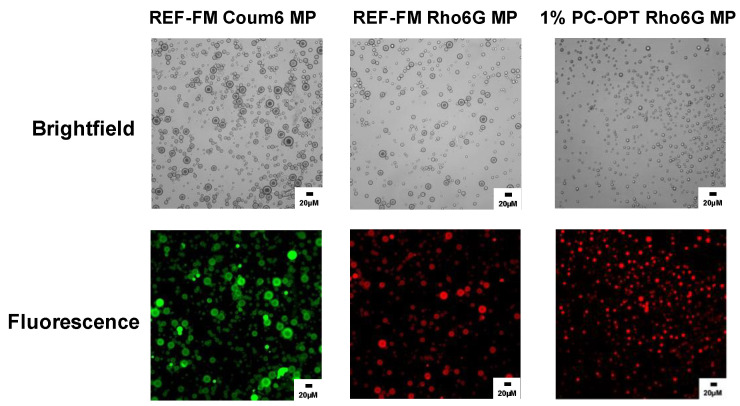
Fluorescence microscopy characterization of dye-loaded PEG-PLGA microparticles.

Consistent with previous findings, these observed differences in drug loading and encapsulation efficiency can be attributed to the distinct physicochemical properties of the dyes [44,45]. The hydrophilicity of rhodamine necessitates enhanced diffusion of the dye out of the emulsion droplets into the external aqueous phase during microparticle production. In contrast, the hydrophobic nature of coumarin facilitates its efficient loading into the hydrophobic PLGA core of the polymer matrix. Thus, these findings underscore the significant influence of the physicochemical properties of biomolecules on their loading efficiency within polymeric carriers [30]. Further characterization of the successful incorporation of these dyes into the microparticles was visually confirmed using a fluorescent microscope (Leica DMI 6000), as shown in Figure 11.

Moreover, given that polymer concentration has been shown to have an impact on encapsulation efficiency, the low amount of encapsulation of rhodamine in the REF-FM may have been caused by the lower polymer concentration of 0.1% *w*/*v* adopted for the reference formulation [6,36]. Likewise, while both formulations of higher polymer concentrations (1% PC-FM and 5% PC-FM) demonstrated an increase in encapsulation efficiency as a result of the availability of more amount of polymer to interact with the dye, there was an apparent reduction in the drug loading as shown in Table 4. This observed difference could be attributed to the possible turbulent effect of higher stir rate and PVA concentration as described in the literature [16,30]. Particularly, an increase in the stirring speed has been reported to generate higher energy in the emulsion, leading to an intensified breakdown of the dispersed droplets forming the microparticles, hence reducing loading efficiency [46]. While higher PVA concentration has been identified with an increase in encapsulation efficiency [30,47], the opposite has been reported for drug loading [29,30,48], as an increase in viscosity due to high PVA concentration may increase the difficulty in achieving ultimate purification and washing of PVA residue, thereby contributing to the mass increase of the particles. Another potential explanation for the significantly lower drug loading observed in the 5% PC-FM formulation could be attributed to the inherently greater mass of the polymer as well as the low initial theoretical drug loading used in this formulation. This is because the % drug loading, calculated as the weight ratio of the loaded drug to the total mass of the drug-encapsulated particles, is often lower for particles prepared with lower initial theoretical drug loading and higher polymer concentrations [36]. As illustrated in Table 4, it is worth noting that loading the same initial drug content into formulations with similar polymer concentrations in the organic phase as the reference formulation but of increased polymer volume (0.01 PVR and 0.02 PVR) resulted in higher encapsulation efficiency as similarly reported in the literature [49]. However, there was a slight reduction in drug loading. This observation supports the idea that increasing the amount of polymer, whether by concentration or volume, allows for more interaction between the drug and the available polymer, leading to enhanced encapsulation. However, the additional mass resulting from the increased polymer quantity may contribute to a slight decrease in drug loading [49].

As indicated in Table 5, we pursued further optimization of drug loading and encapsulation efficiency by elevating the initial theoretical dye loading in the organic phase from 1% *v*/*v* to 5% *v*/*v*. This adjustment resulted in a noteworthy increase in drug loading, measuring at 1.06 ± 0.08 and 2.23 ± 0.33, respectively, for Rho MP and Coum MP, which were synthesized using the reference formulation. However, there was a reduction in encapsulation efficiency, A possible explanation for this occurrence could be the presence of a fixed amount of available polymer to accommodate the loaded drug, thereby resulting in a large amount of dye being unencapsulated due to the inadequacy of available polymer as previously reported [49]. Guided by these findings, we successfully developed a formulation that led to the fabrication of microparticles of similar size to the reference formulation yet exhibiting a 2-fold enhancement in encapsulation efficiency of rhodamine, as shown in Figure 11 and Table 5. This achievement was realized through an initial theoretical loading of 5% *v*/*v* rhodamine dye in a 1% PC-FM while keeping the phase volume ratio constant as the reference formulation. Thus, the microparticles synthesized via this resulting optimized formulation hereafter were referred to as 1% PC-OPT-Rho6G MP.

### 2.7. In-Vitro Release Studies

**Figure 12:**
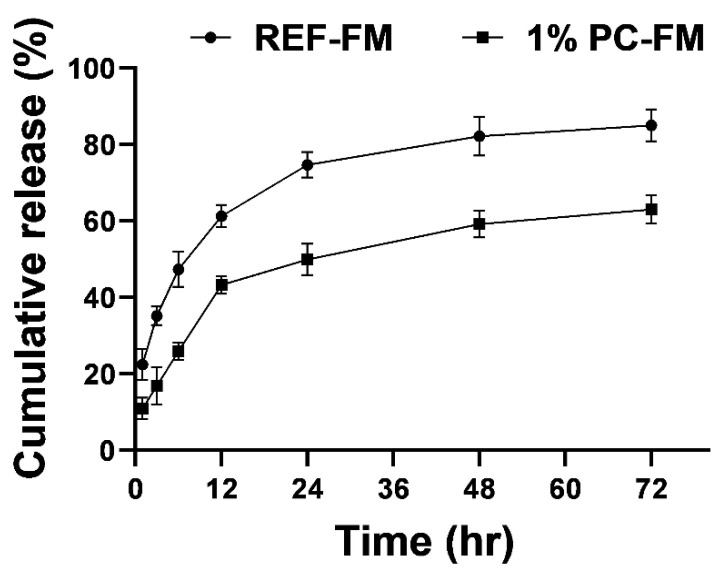
Release profiles of R6G loaded particles from REF-FM and 1%PC-FM in PBS (0.01 M, pH 7.4) under 37 °C shaker incubator (*n* = 3).

The reference formulation and microparticle formulation based on 1% *w*/*v* polymer concentration, hereafter referred to as REF-FM and 1%PC-FM, respectively, were adopted to investigate the release kinetics of R6G from the loaded microparticles in PBS (0.01 M, 7.4 pH). As depicted in Figure 12, all particle formulations displayed an initial burst release pattern, with the formulation based on a 1% *w*/*v* PC-FM exhibiting a minimal level of burst release. This observation could be attributed to a significant quantity of unencapsulated dye adhering to the surface of the REF-FM-MP, primarily due to suboptimal encapsulation when compared to the MPs generated using a 1% PC-FM, a higher polymer concentration. This, in turn, resulted in the excessive initial release upon hydration [50,51,52]. Notwithstanding, both formulations exhibited a gradual and steady release phase, which aligns with the reported release pattern observed in PLGA microparticles. [28,53].

Notably, REF-FM showed a faster release of R6G as compared to 1% PC-FM with a cumulative release of approximately 75% compared to 50% at 24 h, respectively. This finding can be explained based on the slow diffusion in the case of 1% PC-FM, as the dye has to travel a denser pathway from the polymer matrix to reach the dissolution medium [47]. Moreover, the slower hydrolytic degradation of the matrix may also account for the reduced cumulative quantity of drug release from the formulation based on higher polymer concentration since slower water diffusion into the matrix may reduce the accessibility of water molecules to polymer chains. The finding of this observation also emphasizes the feasibility of achieving controlled release in microparticle formulations based on higher polymer concentrations. Additionally, the results presented herein shed light on the distinct impact of polymer concentration on the rate of drug release, as demonstrated through the characterization of rhodamine release from two microparticle formulations of different polymer concentrations but similar particle sizes and distributions. While several studies have reported the influence of polymer concentration on drug release from PLGA particles, it is crucial to acknowledge that these studies often examined drug release using particle formulations of different sizes and distributions, as an increase in polymer concentration generally corresponds to an increase in particle size. Consequently, this results in a dual effect, with both particle size and polymer concentration exerting influence on drug release, owing to the undeniable impact of particle size heterogeneity on drug release from polymeric matrices [9,53,54].

## 3. Materials and Method

### 3.1. Materials 

Poly (ethylene glycol)-methyl ether-*block* -poly (lactide-co-glycolide) (PEG-PLGA_50:50_, Mn_PEG_ 2000 Da and 10,000 Da Mn_PLGA_), dimethyl sulfoxide (DMSO), and coumarin 6 dye were purchased from Sigma Aldrich (St. Louis, MO, USA). Rhodamine 6G dye, dichloromethane (DCM), polyvinyl alcohol (PVA) (87–89% hydrolyzed, high molecular weight), acetonitrile, phosphate buffer saline (PBS), polyvinyl chloride (PVC) tubing, syringes (glass and plastic), syringe needles, and polytetrafluoroethylenes (PTFE) based consumables such as syringe filters (0.45 μm), evaporating dish, and magnetic stirring bars were obtained from Fisher Scientific. All other chemicals and reagents used in this study were of analytical grade. 

### 3.2. Assembly and Assessment of the Coaxial Needle Construct 

**Scheme 1:**
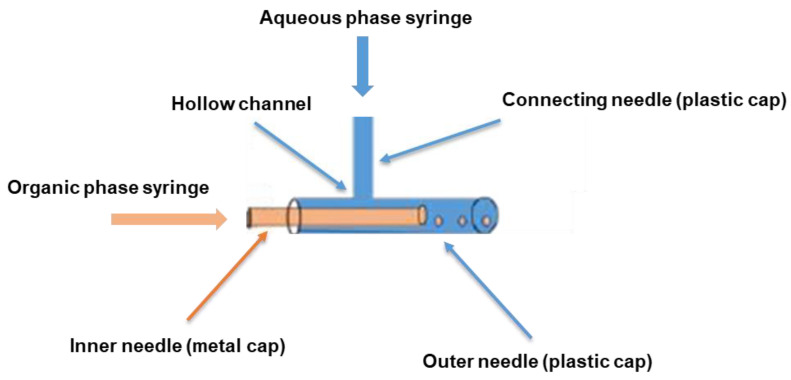
Diagrammatic representation of the coaxial needle assembly.

Typical of all co-flow-based droplet formation devices, the design of our coaxial needle construct was based on a co-flow geometry to facilitate the parallel flow of the dispersed and continuous phases to each other [32,55]. As shown in Scheme 1, the assembly of our simple handmade construct was achieved using three blunt-tip syringe needles with a length of 1.5 inches each. Two of these needles were 16 G needles with plastic-based luer ends, while the third needle was a 30 G Hamilton needle with a luer end made of stainless steel. The plastic base of one of the 16 G needles was punctured and carefully drilled to create a uniformly sized hollow channel using a sharp-tipped 16 G needle to allow the flow of the aqueous phase. Subsequently, the second blunt 16 G needle was inserted gently through the generated hollow channel in the first needle to establish a connection with the first needle. The blunt 30 G Hamilton needle was then inserted centrally along the main axis of the first 16 G needle bearing the orifice. For the purpose of achieving a stable and centered configuration, the resulting assembly was firmly bonded using either parafilm or a suitable adhesive, such as super glue. In this setup, the slender tip of the stainless-steel base of the inner 30 G needle was securely positioned on the wider end of the plastic base of the outer 16 G needle bearing the drilled hollow channel.

To assess the functionality and stability of the coaxial needle construct, appropriate syringes were used to simultaneously flush DCM and DI water through the inner needle and connecting needle, respectively. The efficacy and stability of the construct were verified through the consistent unidirectional flow of the flushing agent via the blunt tip of the outer needle into a collecting beaker. Notably, no discernible instances of leakage or blockages impeding the flow were observed, thus affirming the construct’s effectiveness and stability.

### 3.3. Preparation of PEG-PLGA Microparticles

**Scheme 2:**
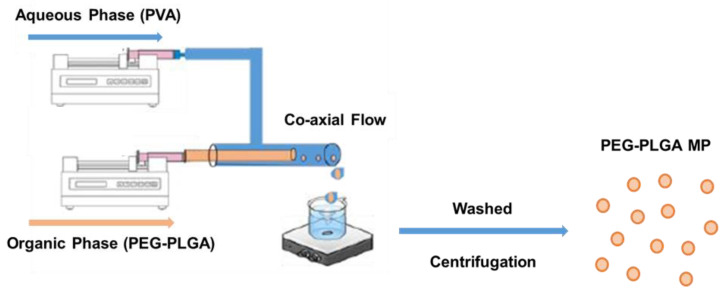
Diagrammatic rendering of the setup used for the microparticle fabrication.

All microparticles were synthesized using a phase separation method based on coaxial needle technology. In detail, the two phases, organic and aqueous, were prepared independently and stored in clean containers. The organic phase consisted of PEG-PLGA polymer dissolved at a desired concentration in dichloromethane (DCM), while the aqueous phase consisted of polyvinyl alcohol (PVA) in deionized (DI) water. The PVA solution was made by dissolving the desired amount of PVA in boiling water to obtain a homogenous solution. Upon cooling, the solution was centrifuged (100× *g*, 5 min) and/or filtered through a 0.45 μm syringe filter with the help of a syringe pump to remove any particulate matter. The glass syringe containing 0.1 mL of organic phase was then placed on a syringe pump (New Era Pump Systems, Inc. in Farmingdale, NY, USA) and connected to the concentrically fixated stainless-steel end of the coaxial needle. A 20 mL plastic syringe filled with aqueous phase was connected to the female luer end of the PVC tubing and placed on a second syringe pump. As illustrated in Scheme 2, the complete assembly of the setup was achieved by connecting the male luer end of the PVC tubing to the plastic end of the coaxial needle gently initiating the co-flow of the two phases into a collecting vessel such as a beaker or PTFE evaporation dish containing 5 mL of PVA solution undergoing continuous stirring at 1000 rpm. Unless stated otherwise, all microparticles were made by setting the flow rate of the co-flow system at 0.1 mL/h for the organic phase and 20 mL/h for the aqueous phase. Following the utmost injection of the volume of the phases, the emulsion was continuously stirred for another hour to ensure complete evaporation of the DCM and solidification of the synthesized microparticle. To remove any residual PVA, the microparticle suspension was collected in a 50 mL falcon tube and washed twice with DI water via centrifugation at (100× *g*, 5 min). Afterward, the microparticles were transferred into a 2 mL microcentrifuge tube and subjected to a final wash before resuspending the eventual microparticle pellet in 0.5 mL of DI water for further studies. For the fabrication of microparticles, we conducted a preliminary analysis of the type of stirrer plate and magnetic stir bar size to be used across all runs. 

. A comprehensive description of the selected process parameters investigated in this study to assess the effect on microparticle sizes and polydispersity is shown in Table 6. Each of these runs involved selectively varying one parameter while maintaining all other fabrication parameters constant in accordance with the reference formulation. The reference formulation was made using 0.1% *w*/*v* of polymer concentration in the organic phase, 5% *w*/*v* surfactant concentration in the aqueous phase, organic-aqueous phase volume ratio (PVR) of 0.005% *v*/*v*, a flow rate of 0.1 mL/h, and 20 mL/h for the organic and aqueous phases and a constant stirring rate of 1000 RPM. 

### 3.4. Preparation of Dye Loaded PEG-PLGA Microparticles

To prepare dye-loaded microparticles, 1 mL of the organic phase consisting of 0.1% *w*/*v* PEG-PLGA in DCM was supplemented with 10 μL of either rhodamine 6G or coumarin 6 dye solution (0.5 mg/mL). The initial dissolution of the dye was performed in DMSO for rhodamine 6G and DCM for coumarin 6 to ensure efficient miscibility and solubility of the dye in the final organic phase.

Following this, 100 μL of the resulting organic phase comprising 1% *v*/*v* of the dye solution was withdrawn using a glass syringe. The microparticle synthesis method here onward was similar to that described above, and the resulting coumarin 6 and rhodamine 6G microparticles hereafter were referred to as Coum6 MP and Rho6G MP. 

The drug loading and encapsulation efficiency were determined by dissolving freeze-dried and pre-weighed dye-loaded microparticles in 1 mL of ethanol for coum6 MP and 1 mL of DMSO for Rho6G MP. The amount of dye release was determined spectrophotometrically at 540 nm (rhodamine 6G) and 460 nm (coumarin 6), respectively [44,56,57], against standard calibration curves of the known concentration of the dyes. Encapsulation efficiency (EE) determination was achieved by the ratio of the mass of encapsulated dye (MD_enc_) to the initial mass of the dye added into the organic phase during the formulation (MD_init_), while drug loading (DL) was calculated as the ratio of the mass of encapsulated dye (MD_enc_) to the total mass of the dried microparticle (MP_tot_) as depicted in the two equations below.

(1) $$EE\%=\frac{{\;MD}_{enc\;}}{{MD}_{init}}\times 100$$

(2) $$DL\%=\frac{{\;MD}_{enc\;}}{{MP}_{tot}}\times 100$$

### 3.5. Characterization of PEG-PLGA Microparticles 

The morphology and size of the microparticles were observed and determined using an inverted microscope (Leica DMI 6000). Briefly, samples were prepared for imaging by pipetting 10 μL of the microparticle suspension in DI water on a microscope glass slide, and images were acquired in brightfield mode using a 20Xobjective. The dye-loaded microparticles were imaged in both brightfield and fluorescent mode. The mean size representing the average diameter of over 200 analyzed microparticles per three independent batches of the same formulation was determined using the Image J (Fiji) open-source image-processing software. The scales of the images were standardized by measuring the distance in pixels of the scale bar provided by the Leica software, inputting the known distance and units, and adjusting the pixel aspect ratio. The ImageJ processed images were measured by zooming into the particles and drawing a diameter line across the microparticles. As an index of the broadness or dispersity of the particle size distribution, the coefficient of variation (CV) of all the analyzed particles was calculated as the ratio of the standard deviation (SD) of the distribution to the mean particle size (SD/mean) as previously reported in the literature [52,58].

### 3.6. In Vitro Release of Rhodamine 6G from Dye-Loaded Microparticles

Freeze-dried R6G-loaded microparticles were suspended in 1 mL of PBS (0.01 M, pH of 7.4) in microfuge tubes. The tubes were then placed in a shaker incubator (C24-New Brunswick Scientific, Edison, NJ, USA) at 37 °C with constant shaking at 90 rpm. Aliquots of 1 mL were collected at regular intervals by centrifuging at 8000 rpm for 5 min and collecting the supernatant as previously described [28]. The microparticles were resuspended in the same amount of fresh PBS. The amount of dye released at each interval in the collected supernatant was analyzed using a UV-vis spectrophotometer (Evolution 60) at 530 nm [57]. The corresponding concentration values were calculated by reference to a standard calibration curve generated in the same release medium. Prior to measurements, instrument calibration and baseline collection were performed. Each microparticle formulation was analyzed in triplicate using a black-walled quartz micro cuvette (0.7 mL, 10 mm). 

### 3.7. Statistical Analysis

The results in this study were analyzed and expressed in terms of the mean ± standard deviation of the distribution using GraphPad Prism 9.5.1 (GraphPad Software, Inc. San Diego, CA, USA). Unless explicitly mentioned, all experiments related to microparticle size measurements were conducted using a minimum of three independent batches. Batch to batch reproducibility of the reference formulation as well as statistically significant differences in microparticles size among various formulation conditions for the same tested process parameter were assessed using Student’s t-test or one-way ANOVA followed by a Dunnett multiple comparison test. A *p*-value less than 0.05 was considered to indicate statistical significance.

## 4. Conclusions

In this study, we report the establishment of a reference formulation based on a combination of individual fabrication and process parameters for the synthesis of uniformly sized microparticles and the subsequent investigation of the impact of process parameters on microparticle characteristics via a modified coaxial flow phase separation method. Among the parameters investigated in this study, stir rate and polymer concentration were identified as the most significant fabrication parameters that influence the size and distribution of microparticles, while surfactant (PVA) concentration was observed as the most substantial parameter that impacts the morphology of the particles. Interestingly, it was observed that the microparticle characteristics were influenced by the fabrication parameters, both individually and when combined with one another. This observation offered the possibility of optimizing microparticle formulations by selecting specific combinations of fabrication parameters, leading to a remarkable twofold increase in the encapsulation efficiency of a model drug without compromising particle characteristics. Hence, the technique described here offers several advantages over methods found in the current literature. It provides flexibility in tuning particle size and morphology with uniformity, enhances consistency in particle reproducibility across batches, allows for scalable production yield, requires less processing steps and time, utilizes simple, inexpensive, readily, and easily made instruments, and facilitates the optimization of drug loading, encapsulation efficiency, and release kinetics for both hydrophobic and hydrophilic drugs.

Altogether, the findings in this study provide a framework for expanding the applicability of this method for synthesizing tunable and scalable polymeric microparticles for drug delivery applications, including the encapsulation of drug molecules for targeted delivery purposes. Moreover, the demonstration of tunability of this method in synthesizing microparticles of different sizes and morphology opens opportunities for further investigation into understanding the interplay between particle characteristics, cellular uptake, and immune cell clearance while also allowing for continuous optimization and improvement of drug loading, encapsulation efficiency, and control over the release of actual drug molecules and biological agents.

## Figures and Tables

**Table 1:** Summary of process parameters affecting PEG-PLGA microparticle size and batch -to-batch reproducibility across three independent batches for each formulation.

Parameter	Formulation	Mean Size (µm ± sd)	%CV
Reference	Reference	6.8 ± 0.1	1.5
Stir Rate	300 rpm	23 ± 1.9	8.3
	600 rpm	11.6 ± 0.6	5.2
	1500 rpm	4.9 ± 0.5	10.2
Polymer (PEG-PLGA) concentration	1% *w*/*v*	12.5 ± 0.6	4.8
	5% *w*/*v*	17.2 ± 1	5.8
Surfactant (PVA) concentration	0.5% *w*/*v*	16.7 ± 0.7	4.2
	2.5% *w*/*v*	7.5 ± 0.3	4
Organic/aqueous phase volume Ratio	0.01% *v*/*v*	8.8 ± 0.4	4.5
	0.02% *v*/*v*	10.6 ± 0.6	5.7
Organic/aqueous phase flow rate Ratio	0.01	7.6 ± 0.4	5.3
	0.025	6.6 ± 0.3	4.5
Organic solvent	25% *w*/*v* ACN/DCM	4.7 ± 0.8	17

Note: Mean size (µm ± sd) was determined by the average of combining the mean values of each independent batch across the same formulation. %CV, calculated as an index of reproducibility, was determined by the ratio of the corresponding SD to the resulting mean expressed as a percentage.

**Table 2:** Scalability and tunability of method via phase volume ratio (PVR) modification.

Formulation	Formulation Parameters	Mean Size (µm ± sd)	CV
REF-FM	0.005 PVR, 5% PVA, 1000 rpm	6.7 ± 3.1	0.45
0.01 PVR-FM	0.01 PVR, 5% PVA, 1500 rpm	5.7 ± 1.5	0.27
0.02 PVR-FM	0.02 PVR, 7% PVA, 1500 rpm	6.1 ± 2.2	0.36

Note: Formulation code: REF FM: reference formulation; 0.01 PVR-FM: 0.01 phase volume ratio formulation, 0.02 PVR-FM: 0.02 phase volume ratio formulation.

**Table 3:** Scalability and tunability of method via increasing polymer concentration.

Formulation	Formulation Parameters	Size (μm)	CV
REF-FM	0.1% *w*/*v* PC, 5% PVA,1000 rpm	6.7 ± 3.1	0.45
1% PC-FM	1% *w*/*v* PC, 5% PVA, 1500 rpm	7.1 ± 2.7	0.38
5% PC-FM	5% *w*/*v* PC, 7% PVA, 1500 rpm	7.2 ± 4.1	0.57

Note: Formulation code: REF FM: reference formulation; 1% PC-FM: 1% *w*/*v* polymer concentration formulation; 5% PC-FM: 5% *w*/*v* polymer concentration formulation.

**Table 4:** Drug loading and encapsulation efficiency of 1% *v*/*v* Coum6 MP and Rho6G MP.

Formulation	Encapsulation Efficiency (%)	Drug Loading (%)
REF-FM with Rho	16.73 ± 0.83	0.43 ± 0.08
REF-FM with Coum	36.33 ± 2.34	0.74 ± 0.09
1% PC-FM with Rho	39.53 ± 5.13	0.21 ± 0.03
5% PC-FM with Rho	58.2 ± 3.99	0.04 ± 0.004
0.01 PVR-FM	20.55 ± 1.07	0.32 ± 0.04
0.02 PVR-FM	23.3 ± 1.14	0.23 ± 0.02

**Table 5:** Drug loading and encapsulation efficiency of 5%*v*/*v* Coum6 MP and Rho6G MP.

Formulation	Encapsulation Efficiency (%)	Drug Loading (%)
REF-FM with Rho	8.97 ± 0.42	1.06 ± 0.08
REF-FM with Coum	24.05 ± 0.54	2.23 ± 0.33
1% PC-OPT FM with Rho	34.6 ± 1.32	0.84 ± 0.02

Note: Formulation code: REF FM: reference formulation; 1% PC-OPT FM: 1% *w*/*v* polymer concentration optimized formulation.

**Table 6:** Process parameters varied in this study.

Parameter.	Condition
Stir rate/speed (rpm)	300, 600, **1000**, 1500
Polymer concentration (% *w*/*v*)	**0.1**, 1, 0.5, 2.5, 5
Surfactant concentration (% *w*/*v*)	0.5, 2.5, **5**
Organic/aqueous volume ratio (% *v*/*v*)	**0.005**, 0.01, 0.02
Organic/aqueous flow rate ratio (mL/h)	**0.005**, 0.01, 0.025
Organic solvent choice (% *v*/*v*)	25% acetonitrile in DCM, **DCM**

Note: The parameters in bold represent the ones used for the fabrication of reference formulation.

## Data Availability

Data sharing is not applicable to this article. All analyzed data is contained within this article or supplementary material.

## Supplementary Materials

The following supporting information can be downloaded at: https://www.mdpi.com/article/10.3390/molecules28186679/s1, Figure S1.1: Comparison of FIMS, 12.7mm-SBL, and 24G-IND to REF-FM; Figure S1.2: Effect of magnetic stirrer type and stir bar length on microparticle size and distributions; Figure S2: Optical micrograph showing the morphological differences of microparticles; Figure S3: Effect of Rho6G and Coum6 loading on mean microparticle size and distribution; Figure S4: Optical micrograph of different size microparticles; Figure S5: Degradation of PEG-PLGA microparticles as examined by their size change over 21 day period; Table S1: Effect of stirrer type, stir bar size, and inner needle diameter on microparticle size and batch-to-batch reproducibility; Table S2: Aspect ratio of the particles; Table S3: Average particle size of microparticles fabricated by varying a combination process parameter.
