# Supplementary material for: Fabrication of PEG-PLGA Microparticles with Tunable Sizes for Controlled Drug Release Application
Source: Molecules. 2023 Sep 18;28(18):6679. doi: 10.3390/molecules28186679 (PMC10534673; doi:10.3390/molecules28186679)
Supplement: Supplementary file 1 [file molecules-28-06679-s001.zip › molecules-2549071-supplementary.pdf]

Supplementary Information:

Fabrication of PEG-PLGA Microparticles with tunable sizes for controlled drug release application.

Paul Nana Kwame Sagoe[1], Eduardo Jose Machado Velázquez [2], Yohely Maria Espiritusanto[1, *], Amelia Gilbert[3], Thalma Orado[1], Qiu Wang[4], Era Jain[1#]

Methods

In vitro stability of PEG-PLGA MP

The stability of the microparticles was assessed over a 21-day period to observe any changes in size and morphology caused by swelling or degradation of the particles over time. Briefly, freshly prepared microparticles based on the reference formulation were characterized immediately after synthesis using an inverted microscope for size and morphology. Subsequently, these particles were suspended in microcentrifuge tubes containing 1 ml of PBS (0.01M, pH 7.4) and maintained in a 37 °C shaker incubator (C24-New Brunswick Scientific) with a constant stirring rate of 100 RPM for further characterization. At specific time intervals, the tubes were withdrawn, subjected to centrifugation (2000 RPM, 5 minutes), and aliquots were taken from the decanted pellets for size and morphology analysis, following the same procedure as described earlier. Following this, an additional 1 ml of PBS was added to resuspend the particles, and the tube was returned to the 37 °C shaker incubator for future time points assessment. Degradation was characterized by observing morphological changes to the particle surface while increase in size was confirmed by calculating the swelling index (SI) of microparticles using equation 1 as similarly reported in literature [1].

$$SI\% = \frac{D_t - D_i}{D_i} \times 100 \quad (1)$$

where D_t represents the diameter attained by microparticles during a set time point of incubation period and D_i is the initial diameter of microparticles at day 0.

Results

S1. Effect of stirrer type and stir bar size on microparticles size and distribution.

The microparticle size and distribution were affected by the type of magnetic stirrer, the size of the stir bar, and the diameter of the inner needle of the coaxial needle construct used in the study. We compared the two different stirrer types, two different stir bar lengths, and two different diameters of the internal needle of the coaxial needle as discussed in the main text section 3.1. The results from these preliminary studies are represented in Table S1 and Figures S1A and S1B.

Table S1: Effect of stirrer type, stir bar size, and inner needle diameter on microparticle size and batch-to-batch reproducibility.

Formulation code	Process Parameter	Mean size (µm ± sd)	%CV

REF- FM	RT Basic series magnetic stirrer<br>Stir bar length: 25.4mm<br>Inner diameter- 30G	6.8 ± 0.1	1.5
FIMS	Fisherbrand Isotemp™ magnetic stirrer	9.3 ± 0.4	4.3
12.7-SBL	Stir bar length -12.7mm	9.1 ± 0.1	1.1
24G-IND	Coaxial needle inner diameter- 24G	8.6 ± 0.9	10.5

Note: Formulation code**:** REF FM: Reference formulation; FIMS: Fisherbrand isotemp™ magnetic stirrer, 12.7-SBL: 12.7mm Stir bar length, 24G-IND: 24G inner needle diameter.

Note: Mean size (µm ± sd) was determined by the average of combining the mean values of each independent batch across the same formulation. %CV, calculated as an index of reproducibility was determined by the ratio of the corresponding SD to the resulting mean expressed as a percentage. %CV= (SD/mean)*100

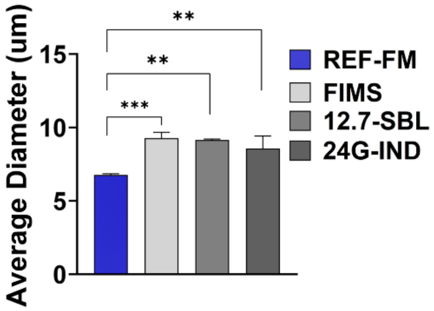

Figure S1.1: Comparison of FIMS, 12.7mm-SBL, and 24G-IND to REF-FM

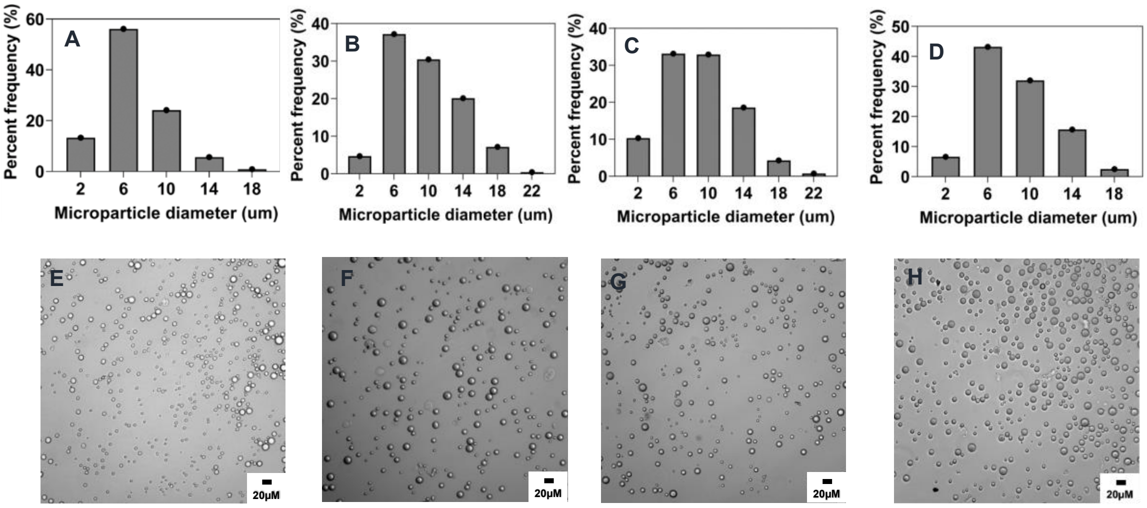

Figure S1.2: Effect of magnetic stirrer type and stir bar length on microparticle size and distributions. Optical micrograph and histogram of particle size distribution: **A&E)** REF-FM **B&F)** FIMS, **C&G)** 12.7mm-SBL and **D&H)** 24G-IND.

S2. Effect of surfactant concentration on microparticle size and shape

We tested a few additional lower and higher concentrations of the surfactant concentration on the size distribution and microparticle size to test the tunability of the method and application for fabricating microparticles with higher polymer concentrations. For this study, we used 1% polymer concentrations. We observed that at lower concentrations of 0.5% PVA, the particle morphology was spherical, and flat with ruffled edges compared to microparticles formed at intermediate concentrations for PVA of 1 and 5% w/v which had smooth and spherical morphology. However, by increasing the PVA concentration further to 10% w/v we obtained a mixed microparticle morphology with some particles assuming an oval shape. The particles have an average aspect ratio of 1.7 showing a longer major axis (**Table S2**). Increasing the PVA concentration also led to a decrease in the particle size as particles made at 0.5% PVA had a mean diameter of 114.3 ± 23.1 while the microparticle made using 1% and 5% PVA w/v had a mean size of 43.9 ± 13 and 24.9 ± 9.9 respectively.

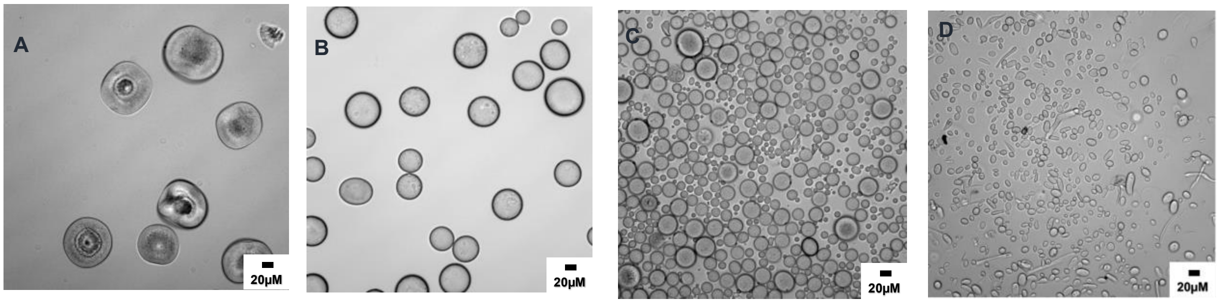

Figure S2: Optical micrograph showing the morphological differences of microparticles synthesized using 1% w/v PEG-PLGA in DCM, 0.005 v/v O/A PVR, 0.005 O/A PFRR, and 600RPM at varied PVA concentration: **A)** 0.5%w/v **B)** 1 %w/v **C)** 5% w/v**, and D)** 10% w/v. PVR: Phase volume ratio, PFRR: Phase flow rate ratio, O/A: Organic/Aqueous

Table S2: Aspect ratio of the particles shown Figure S2 D

Mean Aspect ratio (AR_M)	% of oval MPs (AR >1.1)	% of oval MPs (AR >1.25)	% of oval MPs (AR >1.4)	% of oval MPs (AR >1.6)
1.7 ± 0.4	99	89	75	57

Note: n = 100 particles, AR_M was calculated by averaging the individual AR of 100 particles. Where individual AR was calculated by the ratio of the major axis to the minor axis of the same particle analyzed.

S3. Effect of drug encapsulation on microparticle size

The mean particle size of the microparticle was influenced by the type of the model drug or dye encapsulated in the microparticles. We observed no significant change in microparticle size upon encapsulation of the hydrophilic dye rhodamine 6G (**Fig. S3**) while encapsulation of the coumarin led to a small but significant increase in microparticle size. This may be due to coumarin being more hydrophobic and was encapsulated at much higher efficiency compared to rhodamine thus the significant change in size seen with coumarin.

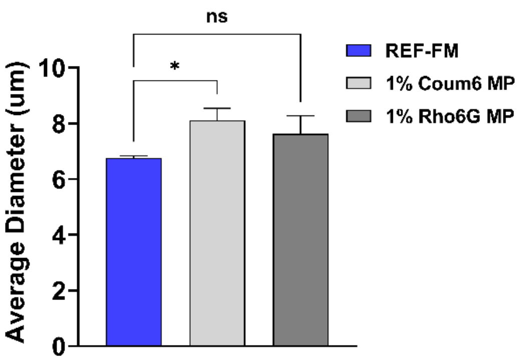

Figure S3: Effect of Rho6G and Coum6 loading on mean microparticle size and distribution.

S4. Effect of varying a combination of different process parameters on microparticle size.

To establish the versatility and tunability of the process we varied a combination of process parameters to obtain a range of microparticle sizes as listed in Table S3. We were to establish that by simultaneously varying the surfactant concentration, PVR, polymer concentration and stir rate

we were further able to expand the range of microparticle sizes that can be obtained by this method to 50-100 μm.

Table S3: Average particle size of microparticles fabricated by varying a combination process parameter. The various formulation parameters are listed below.

**Formulation code**	**Fabrication parameters**	**Size (μm)**	**%CV**
A	2.5%Pol in DCM, 2.5%PVA, 0.005PVR, 0.005PFRR, 300rpm	50 ± 16.5	33
B	5%Pol in DCM, 2.5%PVA, 0.005PVR, 0.005PFRR, 300rpm	76.8 ± 20.8	27.1
C	5%Pol in DCM, 1%PVA, 0.005PVR, 0.005PFRR, 600rpm	102 ± 23.5	23
D	1%Pol in DCM, 0.5%PVA, 0.005PVR, 0.005PFRR, 600rpm	114.3 ± 23.1	20.2

%CV= (SD/mean)*100 calculated based on a single batch formulation of over 100 microparticles sizes analyzed

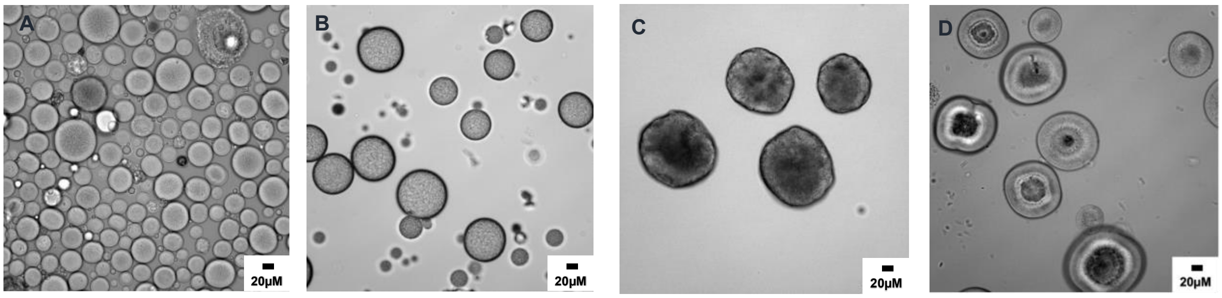

Figure S4: Optical micrograph of different size microparticles synthesized by varying a combination process parameter as indicated for various formulations in Table S3

S5. Degradation of PEG-PLGA microparticles

As shown in **Fig. S5**, optical micrographs revealed that microparticles exhibited a spherical morphology throughout the 21 days observation. However, the onset of degradation led to the decline of particle smoothness, and a corresponding increase in particle size. Particularly, microparticles showed some surface erosion on day 7 with a small but significant increase in size as compared to the initially smooth characterized particles. By day 21, it was evident from the

surface of microparticles that the particles were in the process of fragmentation as shown in **Fig S5**. More also, further characterization revealed an increase in particle size possibly due to swelling, which may be explained by the entrapment of water by the hydrophilic PEG component of the polymer [2]. Notably, the swelling index of microparticles throughout the study was less than 50% despite increasing throughout the 21-study period. This results however is in agreement with reported findings on the swelling index of microparticles less than 15 µm [1].

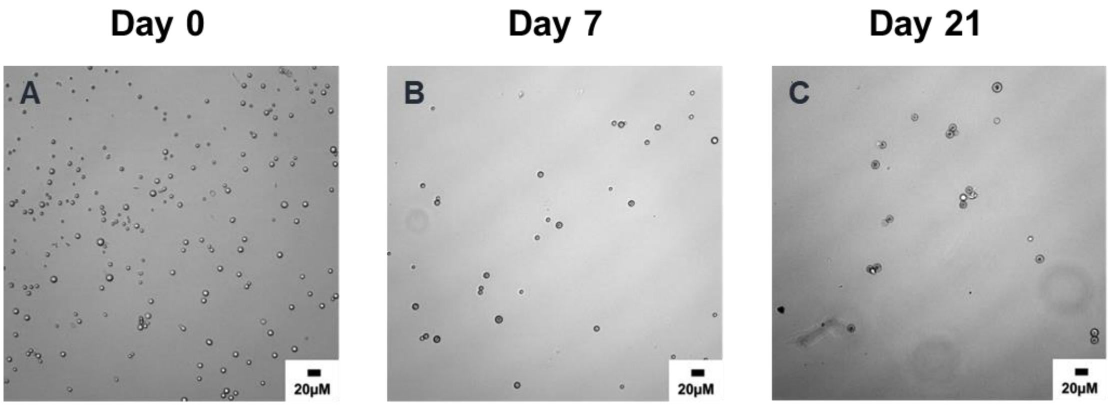

Figure S5: Degradation of PEG-PLGA microparticles as examined by their size change over 21 day period.

**Parameters**	**Day 0**	**Day 7**	**Day 21**
**Mean Size (µm ± sd)**	7.1 ± 1.1	8.0 ±1.7	10.1 ± 2.3
**% CV**	15.5	21.3	22.8
**SI**	0	12.7	42.3

%CV= (SD/mean)*100 calculated based on a single batch formulation of over 100 microparticles sizes analyzed

References

1. Rapier, C.E.; Shea, K.J.; Lee, A.P. Investigating PLGA microparticle swelling behavior reveals an interplay of expansive intermolecular forces. *Scientific Reports* **2021**, *11*, 14512, doi:10.1038/s41598-021-93785-6.
2. Jusu, S.M.; Obayemi, J.D.; Salifu, A.A.; Nwazojie, C.C.; Uzonwanne, V.; Odusanya, O.S.; Soboyejo, W.O. Drug-encapsulated blend of PLGA-PEG microspheres: in vitro and in vivo study of the effects of localized/targeted drug delivery on the treatment of triple-negative breast cancer. *Sci Rep* **2020**, *10*, 14188, doi:10.1038/s41598-020-71129-0.
